# Is There More to Magic Mushrooms than Psilocybin?

**DOI:** 10.1021/acscentsci.5c01146

**Published:** 2025-07-03

**Authors:** Mara Johnson-Groh

## Abstract

Some scientists think that including secondary compounds from psychedelic mushrooms can make for better drugs. With scarce data, others remain skeptical.

## Graphical abstract

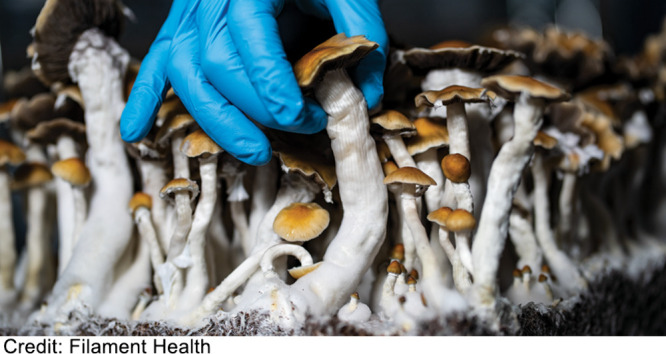

In a suburb of Vancouver, Canada, a nondescript three-story building sits alongside a strip of parking lots. From the outside, it looks like an ordinary commercial office space. But inside is something more extraordinary: rows of shelves stacked with plastic tubs full of magic mushrooms—mushrooms that contain the hallucinogenic chemical psilocybin. In a year, enough psychedelic mushrooms can be produced here to send 80,000 people on hallucinogenic trips.

**Figure:**
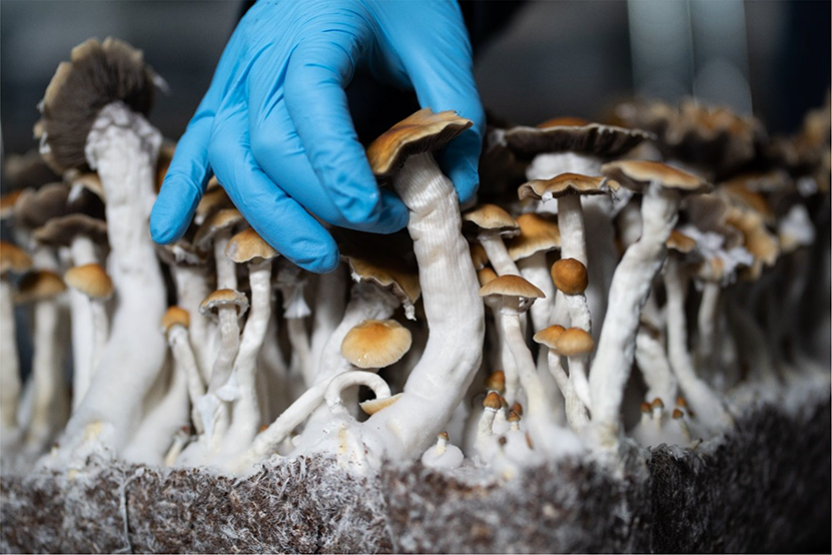
Mature magic mushrooms are carefully harvested to minimize damage. Credit: Filament Health.

Psilocybin is a regulated, illicit substance in most countries, including Canada. But in this facility, run by Filament Health, the mushrooms are not grown for the black market; they are destined for research and clinical trials. These mushrooms could help determine if something important has been missing from psychedelics research.

Psilocybin is a psychedelic compound that, once broken down by the body into psilocin, activates receptors in the brain to unleash a mind-altering experience. After decades of stigmatization, research on psychedelics is finally having a heyday. The research on psilocybin is unveiling its potential to treat challenging mental health conditions like depression, obsessive compulsive disorder (OCD), and stimulant- and opioid-use disorders.

To date, most scientific research on psilocybin has been done with synthetic versions of the compound, not psilocybin from magic mushrooms themselves. Psilocybin was first synthesized in 1958 and synthetic psilocybin has remained the gold standard for testing due to its consistency and cheap production.

But a small group of scientists posit that the magic of these mushrooms is more than their psilocybin alone. Over a dozen different compounds have been identified in magic mushrooms, and some researchers think certain ones could contribute to the duration and intensity of a psychedelic trip, indicators of a phenomenon referred to as the entourage effect.

“Everybody kind of takes it for granted that yes, it’s the psilocybin that produces all these [psychedelic] effects, which probably [for] the large part, that’s true,” says Ryan Moss, chief science officer at Filament Health. “But you have a lot of different compounds in there that can probably aid or modulate the experience.”

Some researchers hypothesize that this entourage effect could also help create better psilocybin-based drugs, with benefits like a reduced chance of a bad trip or an increased trip intensity or duration that could aid treatment. But the data are still scarce, and some scientists are skeptical.

“My unsolicited opinion is that the entourage-effect discussion around *Psilocybe* mushrooms is a bit overhyped and not well supported by solid data,” says chemist Alex Sherwood of the Usona Institute, who has studied compounds in magic mushrooms.

**Figure:**
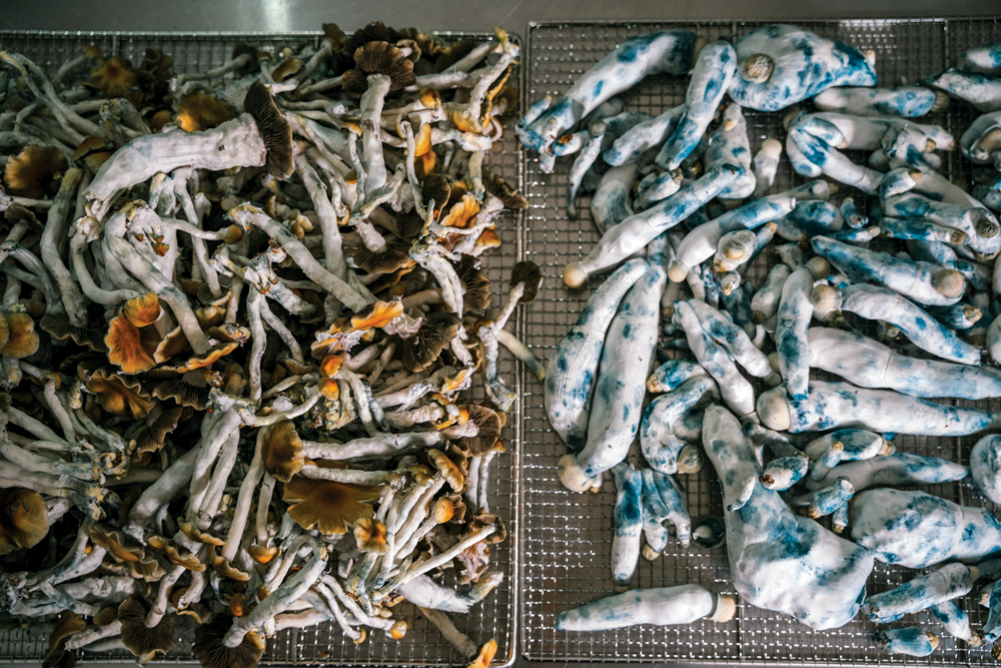
Filament Health has grown many strains of magic mushrooms including two of *Psilocybe cubensis*, shown here. The blue bruising is a result of psilocybin in the mushroom oxidizing. Credit: Filament Health.

Filament Health is hoping to add to the existing data. It is one of just a handful of companies worldwide that are growing magic mushrooms to produce naturally derived psilocybin drugs that retain the secondary compounds found in the mushrooms. Currently, the company is focusing on research and providing its naturally derived psychedelic-drug candidates to dozens of researchers around the world who are rigorously vetting their potential medical merits. Clinical trials with its psilocybin drug are ongoing to test its effectiveness in conditions like major depressive disorder and prolonged grief.

Meanwhile, the results of other research projects on secondary compounds are becoming available. While no human trials directly comparing synthetic and extracted psilocybins have been conducted, chemical, biological, and medical researchers are investigating magic mushrooms’ secondary compounds from multiple angles. The emerging data are painting a complicated picture of a group of mushrooms people have been using for hundreds, if not thousands, of years—and adding fuel to the debate about whether the entourage effect even exists.

## A sum of its parts

Researchers first coined the term entourage effect in 1998 to hypothesize the possible contribution of secondary, pharmacologically inactive compounds in cannabis, upon the discovery of monoacylglycerols that inhibited the metabolism of a cannabinoid. Today the term is being increasingly used in reference to psilocybin products, which, unlike products in the cannabis industry, are dominated by synthetics.

The inclination toward synthetic psilocybin is understandable. Growing *Psilocybe cubensis*, the most cultivated psychedelic mushroom, is not an easy task. Besides all the regulatory hurdles, these mushrooms are highly sensitive to their environmental conditions and susceptible to contamination.

Even in carefully controlled conditions, each individual mushroom develops variations in its psilocybin content and chemical composition. And on top of all those barriers, magic mushrooms contain a mass fraction of only a few percent psilocybin.

These challenges have not discouraged Filament Health. To ensure consistency in dosing, the company goes beyond growing its product. After harvest, its mushrooms are dried in a commercial dehydrator before being crushed into a powder. In a proprietary seven-step extraction process, all the nonactive compounds, like sugars and chitin, are removed. What remains is a concoction of psilocybin and secondary alkaloids that mimics the profile of the original mushroom.

**Figure:**
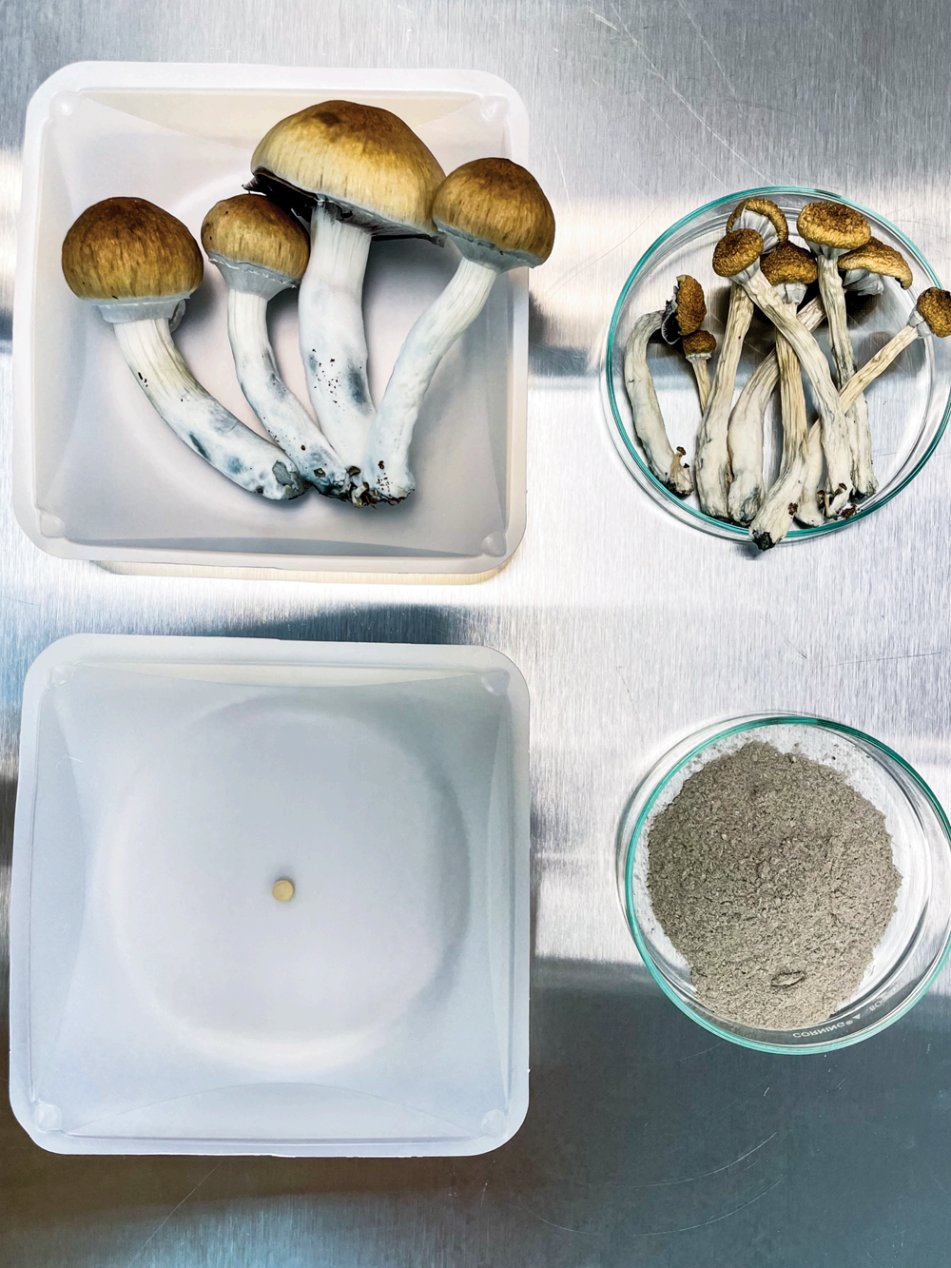
Filament Health creates naturally derived psilocybin drugs (bottom left) over a month-long process that includes growing *Psilocybe cubensis*, drying the mushrooms, and crushing and extracting the psilocybin along with other compounds. Credit: Filament Health.

The process allows Filament Health to ensure an easily testable, consistent, and stable product that can be more readily administered in clinical tests. Moss and his co-workers at Filament Health believe this month-long process is worth the effort because they are strong believers in the entourage effect.

“It’s easy to reduce everything down, and, I mean, as a chemist, I understand,” Moss says. But he thinks the synthetic approach of simply isolating psilocybin is missing a lot of things.

Notably, it misses the dozens of compounds that have been identified in magic mushrooms. Filament Health’s PEX010, the psilocybin drug candidate created from its extraction process, retains 27 different alkaloids.

Whether those compounds are medically important remains to be seen. The ones currently commanding the most attention are the ones that, like psilocybin, are tryptamines: baeocystin, norbaeocystin, and aeruginascin. The compounds are structurally similar to psilocybin, varying by the number of methyl groups pinned onto the molecules.
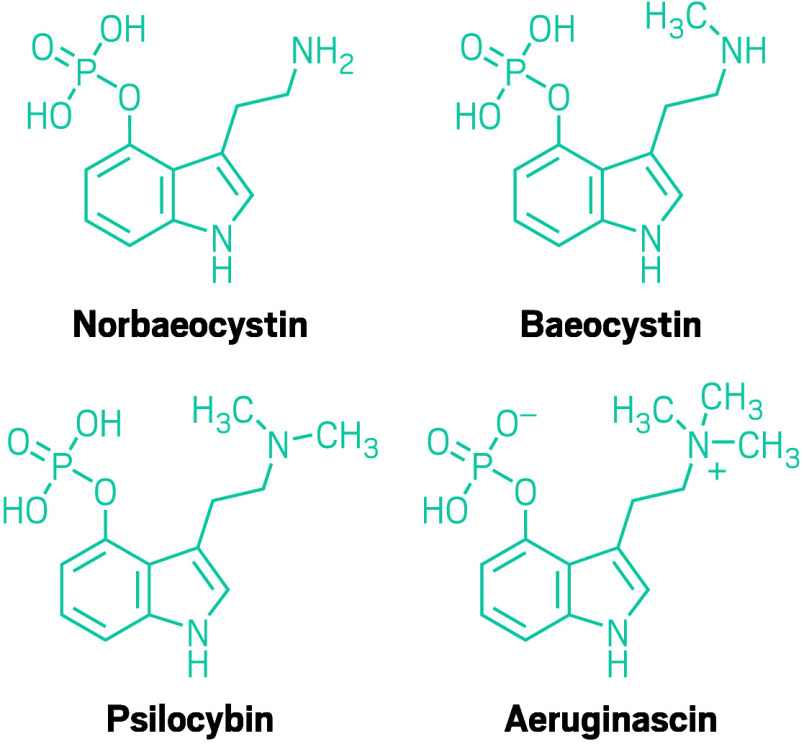


Some research has found that the metabolite of baeocystin, norpsilocin, creates just as strong a response to a key receptor in the brain as psilocin. But other evidence suggests that norpsilocin is too polar to even cross the blood–brain barrier to reach the right neuroreceptor. Concentrations of these tryptamines in magic mushrooms are also significantly lower than the concentration of psilocybin.
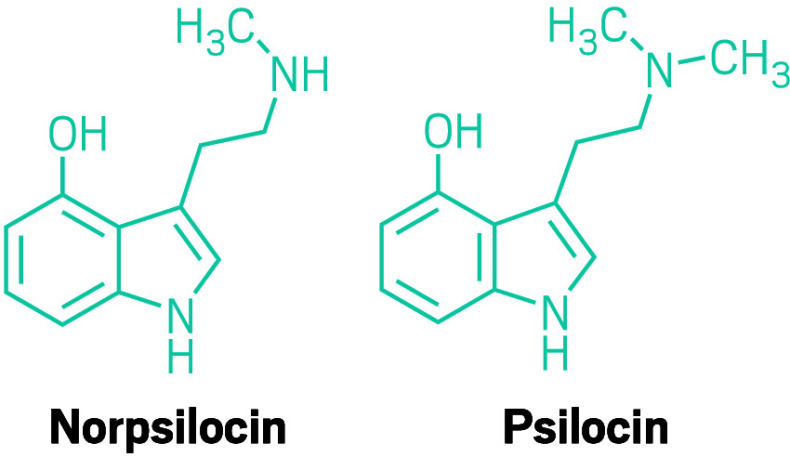


Sherwood, who has studied magic mushrooms’ tryptamines, is skeptical. “The most compelling evidence suggests that compounds like baeocystin and norpsilocin don’t significantly contribute to the psychoactive effects of these mushrooms.”

Beyond tryptamines, researchers have also identified trace amounts of nine terpenes and a half-dozen different β-carbolines, like harmane and harmine. β-Carbolines are thought to play an important role in ayahuasca, a plant-based psychedelic, and some think they could be important in modulating the duration of a magic mushroom trip. But the concentrations of β-carbolines in a typical dose of *P. cubensis* are thousands of times lower than in ayahuasca, leaving some scientists to caution skepticism.

“There’s definitely a chance that certain compounds. . . could change the action of these mushrooms,” says chemist Claudius Lenz of the Helmholtz Centre for Environmental Research–UFZ, who first identified norpsilocin in magic mushrooms. “But I’m more cautious with this entourage effect than many of my colleagues.”

Some of these secondary compounds found in *Psilocybe* mushrooms, like terpenes, have been extensively studied in cannabis. But despite decades of research, the evidence for the entourage effect in cannabis and cannabidiol, more commonly known as CBD, remains scant. A 2020 metastudy concluded that claims of the entourage effect in cannabis are largely unsubstantiated and at times contradictory.

Despite the rocky evidence, a lack of regulation in cannabis has left space for such claims to be widely used in marketing, such as in “whole plant” and “full spectrum” products, which tout therapeutic claims of the entourage effect. Some researchers are concerned that similar claims could abound in the magic mushroom industry even as the science is unsettled.

## The entourage effect under the microscope

The state of research into the entourage effect in cannabis, which spans decades, dwarfs the emerging work being done on the same effect in psilocybin. But that lack of conclusive evidence has not dampened the enthusiasm of many psilocybin researchers. With the increased scrutiny and security on the mushrooms, a more rigorous, ground-up scientific approach is being taken for psilocybin, some say.

“People are so busy making money out of cannabis that they don’t bother to do research with cannabis very much,” says psychiatrist and psychopharmacologist Bernard Lerer of Hadassah BrainLabs, a center for psychedelics research affiliated with Hebrew University. “But there is much, much more fundamental research on psychedelics than there’s ever been on cannabis.”

Anecdotal reports have suggested that magic mushrooms can have a stronger intensity and different visual effects than synthetic psilocybin. Those reports intrigued Lerer, who set out to conduct some of the first animal tests of the entourage effect in magic mushrooms.

In a 2024 study supported by Parow Entheobiosciences, a biotech company that Lerer advises that is developing psychedelic therapeutics, researchers gave one group of mice synthetic psilocybin and others a mushroom extract. By recording the mice’s head twitches, they determined that the drugs induced similarly intense and long-lasting trips. But they found some differences in how the mice’s bodies responded.

When an animal is given a drug, different metabolites accumulate as a result of the drug’s interaction with the body. Predictably, the results showed that the extract, which included a number of compounds in addition to psilocybin, created a larger metabolic response. But more surprising, Lerer says, was the extract’s effect on proteins involved in making new connections between brain cells.

“What we found was that there is a greater production of synaptic proteins following administration of mushroom extract containing psilocybin than there is giving psilocybin alone,” Lerer says. The researchers used Western blot analysis to measure synaptic protein production. “It would appear that mushroom extract increases synaptic plasticity more than psilocybin alone.”

It is well-known that psilocybin promotes neuroplasticity—in fact, this is why some researchers believe psilocybin’s effects can have long-lasting results. But the findings, Lerer says, suggest other compounds are at work.

Some researchers, Sherwood included, remain skeptical. Bryan Roth, an MD and PhD who studies the pharmacology of psychoactive substances at the University of North Carolina at Chapel Hill and was not involved in the study, believes the results need to be verified using complementary, independent methods to measure the proteins.

“The differential effect noted on two synaptic proteins is exceedingly modest,” Roth says, adding that for some protein measurements, no difference was seen. “On the whole, I’m always a bit skeptical of findings from Western blot analysis where very small differences are measured.”

Lerer himself notes that the difference in synaptic protein production seen between the synthetic and extracted forms of psilocybin was not statistically significant. Still, Lerer suspects tryptamines could be responsible for the increase in synaptic proteins; but at this stage, it is just a hunch.

Further differences between mushroom extract and synthetic psilocybin were seen in a second study on mice. Lerer and his colleagues found the mushroom extract produced a stronger effect on reducing behaviors associated with anxiety than did the synthetic psilocybin. But they found mushroom extract and synthetic psilocybin had the same effect on reducing excessive self-grooming, a trait representing OCD. While the study has seen some criticism for its methods and analysis, the findings, if proven, could help demonstrate the potential for targeted strategies using magic mushrooms to treat OCD and anxiety.

Praachi Tiwari, a psychedelics researcher at the Center for Psychedelic and Consciousness Research at Johns Hopkins University School of Medicine, says the studies are interesting and should be followed up on but cautions against directly interpreting the results for clinical use.

“There’s a lot that needs to be done before we can be sure,” Tiwari says.

“We need more comprehensive studies, both preclinical and clinical, in which one tries to tease out the different entourage molecules and find out which of them are making a significant contribution,” Lerer says. “I think that’s the biggest challenge of all.”

## The value in natural

Outside the lab, other researchers are assessing the potential extent of the entourage effect with anecdotal studies. But with few participants and only unblinded assessments, many of these findings are demonstrating a preference for natural products rather than proof of the entourage effect. Nonetheless, this preference is a relevant data point that supports Filament Health’s approach to psychedelics.

**Figure:**
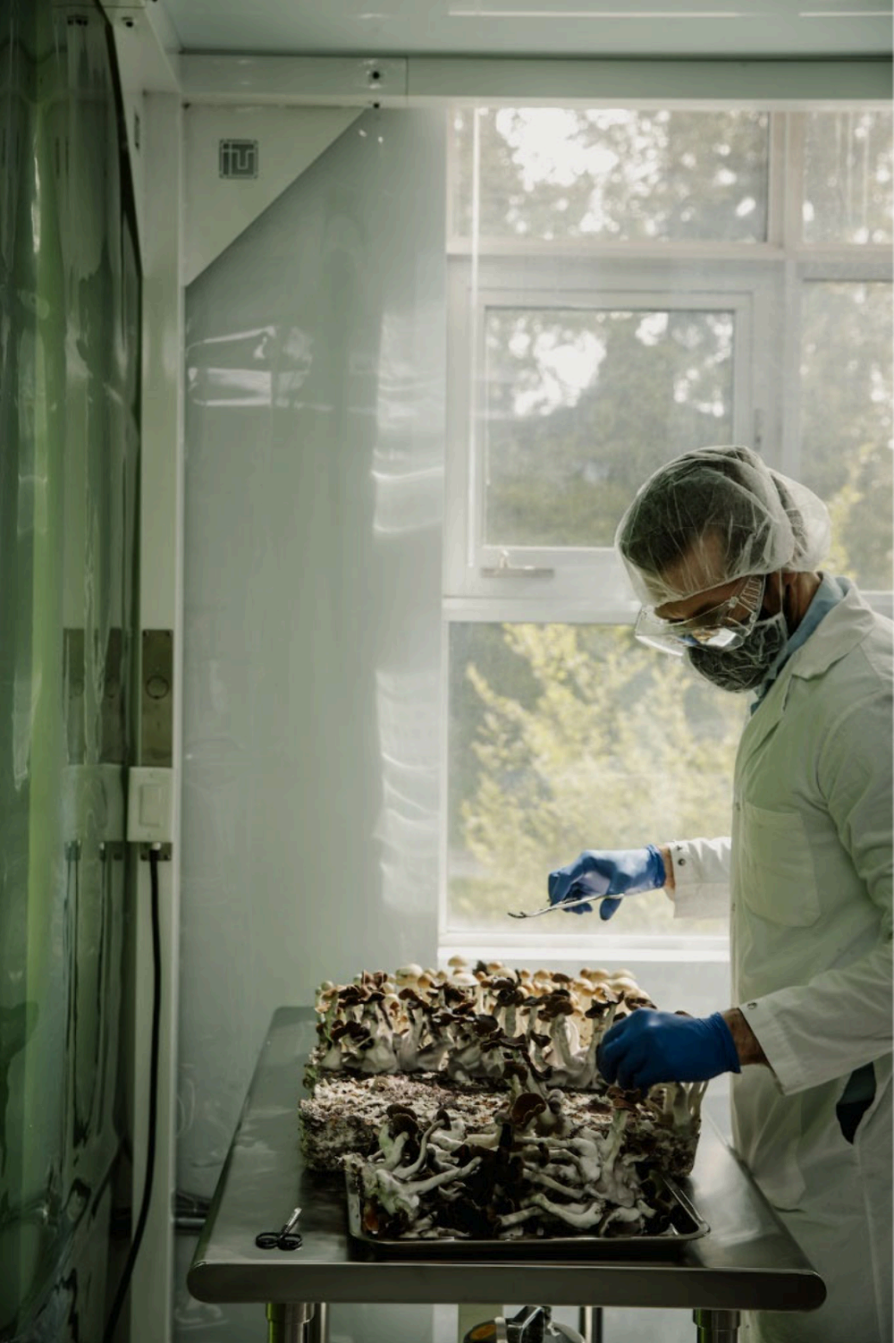
An employee of Filament Health harvests mature magic mushrooms. Credit: Filament Health.

“I think, in general, people’s preferences over the last 20 years has been that natural is better,” Moss says. “We always joke that most people prefer drinking coffee or tea than just taking a caffeine [pill]. And we view this as similar in a way.”

More rigorous anecdotal studies are underway, such as one ongoing survey by Miraculix and the Psychedelic Substances Research Group at the Charité–University Medicine Berlin. These studies, first with user surveys and ultimately with blinded tests, aim to tease out anecdotal differences in trips by accounting for different strains, varying concentrations of active ingredients, prior experiences with psilocybin, gender, and more.

Even if magic mushrooms’ secondary compounds are ultimately not found to have any medical benefits, a natural product could still have value. When psilocybin is administered medically, the patient’s environment and mindset are important in the outcome of the psychedelic trip.

“Having the participant know that this drug you’re taking came from a natural source and was actually grown and things like that might, at least for some people, offer some reassurance in the sometimes challenging experience,” Moss says.

*Mara Johnson-Groh is a freelance contributor to* Chemical & Engineering News, *an independent news publication of the American Chemical Society.*

